# Integrated analyses of host genetics and gut microbiota provide mechanistic insights into feed efficiency in ducks

**DOI:** 10.1186/s40104-026-01410-1

**Published:** 2026-05-19

**Authors:** Haonan Zhao, Xueqin Yang, Xianggui Dong, Junyu Fei, Pengying Li, Lionel Kinkpe, Shuaiqin Wang, Jie Wei, Wei Zhou, Shuisheng Hou, Yunsheng Zhang, Xia Wang

**Affiliations:** 1 College of Animal Science and Technology, Northwest A&F University, Yangling, Shaanxi 712100 China; 2 College of Landscape Architecture and Art, Northwest A&F University, Yangling, 712100 China; 3 State Key Laboratory of Animal Biotech Breeding, Institute of Animal Sciences, Chinese Academy of Agricultural Sciences, Beijing, 100193 China; 4 Chinese Meat Duck Germplasm Innovation Center (Xian County), Xian County, Hebei Province 062250 China; 5 Inner Mongolia Key Laboratory of Duck Breeding, Chifeng, Inner Mongolia 024207 China

**Keywords:** Feed efficiency, Gut microbiota, GWAS, Host genetics, Pekin ducks

## Abstract

**Background:**

Feed efficiency (FE) is a major determinant of productivity, economic sustainability, and environmental impact in livestock production. Although Pekin ducks constitute a substantial proportion of global poultry meat production, improvement of FE has progressed slowly, largely due to an incomplete understanding of its complex biological basis. In particular, how host genomic variation and gut microbial communities—especially across different intestinal segments—jointly contribute to FE remains poorly characterized in ducks.

**Results:**

We integrated whole-genome sequencing (WGS) with segment-resolved microbiota profiles from 313 Pekin ducks to delineate host–microbiota architecture underlying feed conversion ratio (FCR), which exhibited moderate heritability ( *h*^2^=0.23,* P*_LRT_ = 2.53 × 10^–2^). Genome-wide association study (GWAS) identified two major genomic regions involving two genes *ALG13* and *TRPC5*, which implicated in appetite regulation and energy balance to impact FE. Meanwhile, gut microbial communities exhibited spatial heterogeneity along the intestinal tract, with host genetics-associated taxa such as *Solobacterium* (cecum) and *Eubacterium_eligens_group* (jejunum) negatively correlating with FCR, likely via enhanced short-chain fatty acid production and improved energy harvest. Variation partitioning revealed that host genetics explained the largest FCR variance (36.96%), followed by cecal microbiota.

**Conclusions:**

Our results highlight coordinated host–microbiota interactions influencing FE, offering targets for breeding and microbiota-based interventions.

**Supplementary Information:**

The online version contains supplementary material available at 10.1186/s40104-026-01410-1.

## Background

Meeting the global demand for animal protein in a sustainable and efficient manner is a paramount challenge for modern agriculture [1]. Central to this challenge is feed efficiency (FE)—the ability of an animal to convert feed into body mass—as feed constitutes the largest operational cost in livestock production [2–4]. Improving FE is therefore critical for enhancing economic viability and reducing the environmental footprint of the industry. In poultry, ducks represent a significant portion of global meat production, with Pekin ducks being the primary breed due to their rapid growth, resilience, and high survival rates [5–7]. Despite these advantages, the Pekin duck industry faces persistent challenges in further improving FE, making it a key priority for breeding programs.

Feed efficiency is a critical economic trait in animal production [8, 9]. One of the most commonly used metrics to evaluate FE is the feed conversion ratio (FCR, or feed-to-gain ratio) [10], which measures the amount of feed consumed per unit of weight gain over a specific period [11]. Due to its simplicity and direct relationship between input costs and output returns, FCR is widely used to characterize effectiveness of dietary across species [12–15].

As a complex trait, FCR is influenced by various factors, among which animal genetics play an important role. Numerous studies have reported a moderate heritability (0.18–0.39) for FCR in animals [15–20], indicating a significant level of genetic control. Genome-wide association studies (GWAS) have successfully identified quantitative trait loci (QTLs), single nucleotide polymorphisms (SNPs), and candidate genes associated with FCR. For instance, in laying-type chickens, the *SUCLA2* gene was linked to FCR, potentially by enhancing energy metabolism [18]. In Pekin ducks, genes such as *SCTR*, *LACTB2*, *SLC35F1*, *SLC6A2* and *DDX10* have been implicated in ileal development and feed consumption [21]. Similarly, genes like *MT3* in large white pigs and *PPM1E* in sheep have been identified as key regulators influencing growth and metabolic homeostasis to affect FE, respectively [22, 23].

Another key factor affecting FCR is the gut microbiota [24], as its composition and activity influence the host's ability to digest feed and extract energy [25]. Specific microbial taxa have been consistently associated with FE. For example, supplementation with *Bacillus subtilis* in broilers improves nutrient digestibility and reduces FCR [12, 26]. In Muscovy ducks, drinking the water with duck-derived lactic acid bacteria (DDL) significantly reduced the FCR by shaping the gut morphology, microbiota and metabolism [27, 28]. In pigs, *Christensenellaceae*, efficient polysaccharide degraders, are often enriched in animals with higher FE, as they produce short-chain fatty acids (SCFAs) that serve as an important energy source for the host [29].

Furthermore, evidence shows that host genetics can shape the gut microbiota, and that the two factors jointly regulate complex traits like FE [24]. For example, heritable microbes like *Parabacteroides* and *Megasphaera* in chickens, and specific *Bacteroides* and *Prevotella* taxa in pigs, are linked to host genetic loci and contribute to energy harvest through SCFA production, thereby improving FE [9, 30]. Similarly, Li et al. [31] identified 14 heritable rumen microbial taxa significantly associated with host FE in cattle. These taxa include genera such as *Butyrivibrio*, *Ruminococcus*, and *Christensenellaceae R-7 group*, which are known for fiber degradation and SCFA production. Despite these advances, most studies in Pekin ducks have investigated host genetics and gut microbiota in isolation, leaving their combined contributions to FE largely unexplored.

In this study, we integrated host genetic data with microbial profiles from four intestinal segments (duodenum, jejunum, ileum, and cecum) of 313 Pekin ducks to elucidate spatial host–microbiota interactions, evaluate their combined effects on FE, and identify specific genetic variants and microbial taxa significantly associated with this trait. These insights will not only deepen our understanding of the biological mechanisms underlying FE, but also provide a theoretical foundation for developing sustainable strategies to improve FE in Pekin ducks.

## Methods

### Animal feeding and phenotype data collection

A total of 313 male Pekin ducks, comprising two lines of ducks with divergent FE (151 ZF ducks with high FE and 162 CF individuals with low FE), were used in this study. All ducks were hatched on the same day and raised individually in single cages equipped with nipple drinkers and tubular feeders until 42 days of age at the Beijing Duck Breeding Farm of the Institute of Animal Sciences, Chinese Academy of Agricultural Sciences, Beijing, China. The ducks feeding management methods and composition of the commercial diet were detailed in our previous research [32].

The total feed intake (FI) and body weight gain (BWG) of each duck were recorded and calculated from 22 to 42 d of age. According to the measured data, the feed conversion ratio (FCR) was calculated referring to our previous method [32]. The detail formula is shown as follows:

$$FCR=FI/BWG$$

Normality for FCR was checked using the Shapiro–Wilk test in the R program (version 4.4.1).

### Blood and gut segment sample collection

At the age of 42 d and after 12 h fasting, whole blood samples were obtained from the wing veins of 313 ducks. After centrifugation at 3,500 × *g* for 5 min at 4 °C, the plasma was obtained and then stored at −20 °C until use. Then all the ducks were euthanized using gas inhalation via carbon dioxide and four segment contents (duodenal-D, jejunal-J, ileal-I, and cecal-C) were collected according to the methods of our previous study [33].

### Host and gut microbial DNA extraction

The host genomic DNA was isolated from blood samples using the TIANamp Animals DNA extraction Kit (TIANGEN, Beijing, China). The gut segment samples were used for the gut microbial DNA extraction by EasyPure^®^ Genomic DNA Kit (TransGen Biotech, Beijing, China). Then the concentration and purity of DNA was measured by a Nanodrop instrument (Thermo Fisher Scientific, Waltham, MA, USA) and the quality of DNA was assessed on 1% agarose gel electrophoresis to ensure successful DNA isolation. Finally, a total of 313 host DNA and 1,252 microbial DNA samples were used for sequencing.

### Whole-genome sequencing (WGS) and data processing

After passing quality control, the host DNAs were sheared by sonication, purified, end-repaired, dA-tailed, and ligated to Illumina paired-end adaptors. Then the fragment size selection is performed using agarose gel electrophoresis, followed by PCR amplification to construct the sequencing library. Based on quality-approved libraries, whole-genome resequencing was performed on the Illumina HiSeq 2500 Sequencer (Illumina, Inc., San Diego, CA, USA) to generate 150 bp paired-end reads of tenfold depth.

To reduce mapping errors, quality control was performed via fastp software (version 0.23.4) [34] with default parameters to remove low-quality reads. The obtained clean reads from each duck were aligned to the Pekin duck reference genome (IASCAAS_PekinDuck_T2T, https://www.ncbi.nlm.nih.gov/datasets/genome/GCF_047663525.1/) using the BWA tool (version 0.7.17) [35] with default parameters. Then the potential PCR duplicate reads were removed from the alignment results by the Picard toolkit (version 3.1.1, https://broadinstitute.github.io/picard/) and indexed by the SAMtools software (version 1.19) [36]. The variants detection was performed using the modules in GATK software (version 4.0.5.1, https://github.com/broadinstitute/gatk/). Specifically, gVCF files were first generated for each sample using HaplotypeCaller and CombineGVCFs modules, followed by genotypes calling with GenotypeGVCFs module. After merging the VCF file of SNPs by modules MergeVcfs and SelectVariants, SNPs were filtered via the VariantFiltration module, applying the following criteria: (1) QD < 2.0, (2) MQ < 40.0, (3) FS > 60.0, (4) SOR > 3.0, (5) MQRankSum < −12.5, and (6) ReadPosRankSum < −8.0. Subsequently, only biallelic variants were used to perform more robust quality control by PLINK software (version 1.9, https://www.cog-genomics.org/plink/) with the criteria as follows: --geno 0.1, --maf 0.05, --hwe 0.000001. Finally, the remained SNP dataset was subjected to genotype imputation based on the BEAGLE (version 5.3) [37]. After these procedures, a total of 10,159,633 SNPs, distributed across 39 autosomal chromosomes and 313 Pekin ducks, were obtained for subsequent analyses.

### 16S rRNA gene sequencing and analysis

The 16S rRNA gene sequencing and data basic processing was performed according to the methods described in our previous study [33], that the taxonomic classification was performed based on the SILVA reference database (v138). To focus on the core gut microbiome and minimize potential sequencing errors, we retained ASVs with total relative abundance ≥ 0.05% and detected in ≥ 30% of samples. The prevalence threshold of 30% falls within the recommended range (20%–70%) for core microbiome identification [38], while the abundance threshold of 0.05% (> 10 copies) is applied to filter out low-abundance taxa that may arise from sequencing artifacts [39]. Based on the filtered ASVs table, alpha-diversity metrics and beta-diversity metrics were further calculated based on the methods described in our previous study [33]. To determine the potential origins of the gut microbiota, we estimated the contribution of different source environments for cecum microbiota using the microbial source tracking method FEAST [40] (https://github.com/cozygene/FEAST) implemented in R program. All relative abundances of bacteria were then centered log-ratio (CLR) transformed by the compositions (version 2.0-9, https://cran.r-project.org/web/packages/compositions/) package in R program.

### Construction of host genetic relationship matrix and microbial relationship matrix

Based on the independent dataset of 10,159,633 SNPs, we estimated the principal components (PCs) and genetic relationship matrix (GRM) of 313 individuals based on the method proposed by Yang et al. [41], with the formula as follows:

1 $${g}_{ij}=\frac{1}{n}{\sum }_{z=1}^{n}\frac{({x}_{iz}-2\overline{{p }_{z}})({x}_{jz}-2\overline{{p }_{z}})}{2\overline{{p }_{z}}(1-\overline{{p }_{z}})}$$

where ${g}_{ij}$ represents the estimated genetic relationship between ducks *i* and *j*; *n* means the number of variants, *x*_*iz*_ and *x*_*jz*_ are the counts of reference alleles in ducks *i* and *j*, respectively; $\overline{{p }_{z}}$ stands for the frequency of reference allele in the duck population.

Afterwards, the microbial ASVs from four gut segments that passed QC were normalized (z-value) and used to constructed the microbial relationship matrix (MRM) based on the equation described by Wen et al. [42]. The equation is shown as follows:

2 $${m}_{sij}=\frac{1}{{n}_{s}}\sum_{o=1}^{{n}_{s}}\frac{({x}_{sia}-\overline{{x }_{sa}})({x}_{sja}-\overline{{x }_{sa}})}{{\sigma }_{sa}^{2}}$$

in which *m*_*sij*_ stands for the estimated microbial relationship in site *s* between ducks *i* and *j*; *n*_*s*_ represents the total number of ASVs in site *s* used for the relationship calculation;* x*_*sia*_ and *x*_*sja*_ are the relative abundances of ASV *a* in site *s* of ducks *i* and *j*, respectively; $\overline{{x }_{sa}}$ is the average relative abundance of the ASV *a* in site *s* in the duck population; and ${\sigma }_{sa}^{2}$ is the variance of the abundance of ASV *a* in site *s*.

### Investigating the effects of host genetics on FE

To assess the contribution of host genetics on FE, we estimated the SNP-based heritability (${h}^{2}$) of the FCR by restricted maximum likelihood (REML) analysis implemented in the GCTA software (version 1.94.1, https://yanglab.westlake.edu.cn/software/gcta/). The estimation model is shown as follows:

3 $$\boldsymbol y\;=\;\boldsymbol Z\boldsymbol a\;+\;\boldsymbol g\;+\;\boldsymbol e$$

in which ${\boldsymbol{y}}$ represents the vector of observations for FCR; ${\boldsymbol{a}}$ is a vector of fixed covariates with the corresponding design matrix $\boldsymbol Z$, including the top five host genetic PCs; ${\boldsymbol{g}}$ is the polygenic effects that follows the normal distribution of N(0, $\boldsymbol G\sigma_a^2$), where $\boldsymbol G$ is the GRM and ${\sigma }_{a}^{2}$ is the polygenic additive variance; ${\boldsymbol{e}}$ is a vector of residual effect following a distribution of N(0, $\boldsymbol I\sigma_e^2$), where ***I*** is an identity matrix and ${\sigma }_{e}^{2}$ is the residual variance. Heritability (${h}^{2}$) is calculated as ${h}^{2}={\sigma }_{g}^{2}/{\sigma }_{p}^{2}$, where ${\sigma }_{p}^{2}$ represents the phenotypic variance. The likelihood ratio test (LRT) *P*-value was calculated to examine the significance of ${h}^{2}$ estimation. Later, GWAS was conducted to detect the host genetic variants related to FE using the GEMMA software (version 0.98.5, https://github.com/genetics-statistics/GEMMA). The SNPs located on the sex chromosomes were removed to minimize potential sources of bias, and the top five PCs were added as covariates to the GWAS estimation mixed linear model (MLM) as follows [9]:

4 $${\boldsymbol{y}}=\boldsymbol{Z}{\boldsymbol{a}}+{\boldsymbol{X}}b+{\boldsymbol{g}}+{\boldsymbol{e}}$$

In the above formula, ${\boldsymbol{X}}$ is a vector of allele counts (0, 1, 2); $b$ represents the SNP effect; the other parameters were same as described in formula (3). Genome-wide and suggestive significance thresholds were determined referring to the modified Bonferroni correction method [43], yielding 4.92 × 10^−9^ (0.05/10,159,633) and 9.84 × 10^−8^ (1/10,159,633), respectively. Subsequently, we annotated the significant SNPs using the ANNOVAR software (https://annovar.openbioinformatics.org/) [44]. Evaluation and visualization of linkage disequilibrium (LD) levels among the SNPs significantly associated with FCR was completed using LDBlockShow software (version 1.41, https://github.com/BGI-shenzhen/LDBlockShow). The LD coefficient *r*^2^ was calculated as an indicator of LD strength. Haplotype blocks were delineated based on the criterion that adjacent SNPs maintained a pairwise *r*^2^ > 0.8 across a continuous genomic region.

### Detecting the influence of host genetics on the gut microbiota

To detect the influence of host genetics on the gut microbiota, we first performed the heritability estimation of four gut segments microbial heritability (${h}^{2}$) at the genus level based on the same model as model (3), but $\boldsymbol{y}$ in the model represents the vector of observations for microbial features in each gut segments now. The microbial genera with ${h}^{2}$ more than 0.2 and *P*_LRT_ less than 0.05 was identified as heritable genera. Afterwards, the microbiome genome-wide association studies (mGWAS) of heritable genera were performed based on the same model as model (4), except that the meaning of $\boldsymbol{y}$ was replaced by the vector of microbial feature observations in each gut segments. Genome-wide and suggestive significance thresholds were set equally to those used in examining the significance of SNP-FCR associations. SNP annotation was also performed by ANNOVAR software (https://annovar.openbioinformatics.org/) [44].

### Identification of FCR-associated microbiota affected by host genetics

Using the filtered genus-level microbiota dataset, associations between qualified taxa and FCR were analyzed by microbiome-wide association studies (MWAS) based on a two-part model implemented in a customized R script, which was described in previous studies [9, 45, 46]. The first part of the model addressed binary traits, defined by the presence (abundance > 0, coded as 1) or absence (abundance = 0, coded as 0) of each genus. The second part focused on quantitative traits, applying regression analyses between phenotypes and microbial relative abundances. The MWAS model is described as follows:

5 $$\bf \bf {\boldsymbol{y}}=\left\{\begin{array}{c}{\beta }_{1}\boldsymbol{b}+{\boldsymbol e}\\ {\beta }_{2}\boldsymbol{q}+{\boldsymbol e}\end{array}\right.$$

in which ${\boldsymbol{y}}$ denotes the vector of FCR observation; $\boldsymbol{b}$ is the binary trait of a specific microorganism; $\boldsymbol{q}$ is the log_10_-transformed relative abundance of that microorganism; ${\beta }_{1}$ and ${\beta }_{2}$ represent the regression coefficients for the binary and quantitative models, respectively; and ${\boldsymbol{e}}$ is the intercept. The second part of the model was applied only to present microorganisms, with *P*-values from both parts adjusted using the Benjamini-Hochberg (BH) method. The taxa with an FDR < 0.05 in either part was considered as key microbiota.

Afterwards, FCR-associated SNPs were selected, and differences in microbial abundances across the four gut segments among ducks with different genotypes were assessed by one-way analysis of variance (ANOVA) and the Wilcoxon rank-sum test. Then we integrated the taxa from MWAS results, taxa with mGWAS-identified SNPs, and taxa showing significant differences across genotypes at FCR-associated SNPs, and obtained the FCR-associated microbiota affected by host genetics.

### Calculation of multi-omics-explainability for FCR

To clarify the multi-omics-explainability for FCR, we utilized the GRM and MRM to construct the MLM model using the sommer package (https://covaruber.r-universe.dev/sommer) in R program. The detail of MLM was as follow:

6 $$\boldsymbol y=\boldsymbol Z+\boldsymbol g+{\boldsymbol m}_d+{\boldsymbol m}_j+{\boldsymbol m}_i+{\boldsymbol m}_c+\boldsymbol e$$

where ${\boldsymbol{y}}$ denotes the vector of FCR observation; ${\boldsymbol{Z}}$ is the fixed effect; ${\boldsymbol{g}}$ has the same meaning as that in model (3); ${{\boldsymbol{m}}}_{{{d}}}$, ${{\boldsymbol{m}}}_{{{j}}}$, ${{\boldsymbol{m}}}_{{{i}}}$, and ${{\boldsymbol{m}}}_{{{c}}}$ are the microbial random effects of four gut segments, respectively; and ${\boldsymbol{e}}$ is the residual effect. For each intestinal segment $k\in \left\{d,j,i,c\right\}$, the random effect was assumed to follow ${{\boldsymbol{m}}}_{{{k}}}\sim N(0,{ {\boldsymbol{M}}}_{k}{\sigma }_{{m}_{k}}^{2})$, in which ${\boldsymbol M}_k$ is the microbial relationship matrix of segment $k$, and ${\sigma }_{{m}_{k}}^{2}$ is the corresponding microbial variance. *P*-values for the omics-explainability estimations were obtained from LRT of the random effects, with significance defined as *P* < 0.05.

## Results

### Characterization of host phenotype and sequencing data

**Fig. 1:**
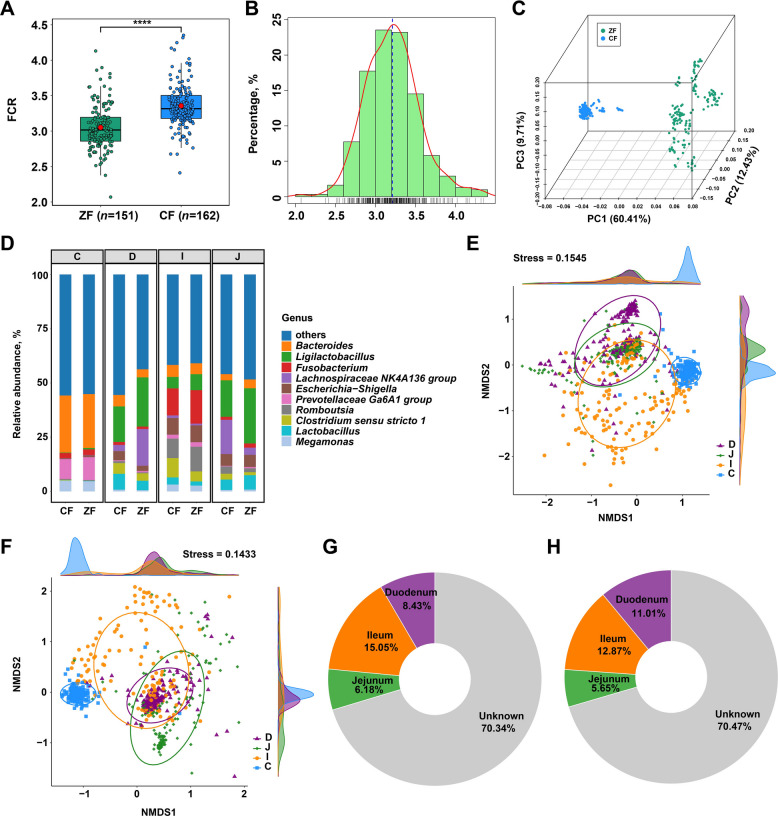
Phenotypic distribution and population structure of Pekin ducks based on feed efficiency. **A** Comparison of FCR value between ZF and CF groups (^****^P < 0.0001). Green spots denote the ZF ducks, and blue spots represent the CF ducks. The red spots in two groups denote the average of FCR in each group. **B** Frequency distribution of FCR across all individuals. **C** Three-dimensional PCA based on whole-genome SNP data. Green spots denote the ZF ducks, and blue spots represent the CF ducks. **D** Composition of intestinal microorganisms at the genus level in ZF and CF ducks. **E** NMDS at the genus taxonomic level in ZF ducks. **F** NMDS at the genus taxonomic level in CF ducks. **G** Microbial source tracking of cecum in ZF ducks. **H** Microbial source tracking of cecum in CF ducks

To characterize phenotypic variation in Pekin ducks, we first compared FCR values between the two groups (ZF with high FE and CF with low FE). As illustrated in Fig. 1A, the ZF group (*n* = 151) exhibited a significantly lower FCR than the CF group (*n* = 162) (*P* < 0.0001), demonstrating marked differences in FE between the two populations. Across all the ducks, FCR values approximated a normal distribution (Fig. 1B), supporting suitability for subsequent analysis. To acquire comprehensive host genomic information, WGS was performed on all individuals at approximately 10 × coverage. This generated a total of 3.4 Tb of high-quality paired-end clean data, with an average genome coverage of 96.15% per individual, thereby enabling high-confidence identification of host genetic variants. Following stringent quality control, a final set of 10,159,633 SNPs distributed across 39 autosomal chromosomes was retained for downstream analyses. Principal component analysis (PCA) based on the genome-wide SNP dataset revealed a clear separation between the two groups along the first principal component (PC1), which explained 60.41% of the total genetic variance (Fig. 1C), further confirming distinct genetic backgrounds between ZF and CF ducks.

Additionally, the 16S rRNA sequencing was conducted on four intestinal segments (duodenum-D, jejunum-J, ileum-I, and cecum-C), yielding a total of 347,410,739 quality-filtered clean reads, thus providing comprehensive microbial community profiles. After reads splicing and quality filtering, ASVs were clustered with 100% sequence identity, and subsequently classified into 83 phyla, 193 classes, 450 orders, 683 families, 2,412 genera, and 3,108 species, representing a broad microbial diversity across the intestinal tract. The microbial community composition showed distinct taxonomic patterns at the genus level across the four intestinal segments between two groups (Fig. 1D). *Ligilactobacillus* was most abundant in duodenum and jejunum, while *Fusobacterium* and *Bacteroides* dominated the ileum and cecum, respectively. Meanwhile, variations in the relative abundances of these microorganisms across intestinal segments were observed in ZF and CF ducks, suggesting that feed efficiency may be associated with specific microbial compositional changes along the intestinal tract. Non-metric multidimensional scaling (NMDS) analysis further revealed distinct clustering patterns of the gut microbial communities along the intestinal tract in both groups (Fig. 1E and F). Samples from the small intestine (duodenum, jejunum, and ileum) and cecum were clearly separated, and the degree of dispersion for samples in the small intestine is higher than in the cecum, indicating substantial compositional differences among these regions. Moreover, noticeable differences in microbial community composition were also observed between the two groups within each intestinal segment. Microbial source-tracking analysis revealed that the cecal microbiota primarily originated from the ileum, contributing 15.05% in ZF and 12.87% in CF ducks (Fig. 1G and H). The duodenum and jejunum contributed 8.43% and 6.18% in ZF, and 11.01% and 5.65% in CF, respectively. However, a large proportion of cecal taxa (70.34% in ZF and 70.47% in CF) remained of unknown origin, indicating the presence of a highly distinct and potentially specialized microbial ecosystem in the Pekin duck cecum. Together, these datasets provide high-resolution host genomic and gut microbial profiles, enabling robust investigation into the contributions of host genetics and microbiota to feed efficiency in Pekin ducks.

### Genome-wide association study reveals novel loci controlling feed efficiency in Pekin ducks

**Fig. 2:**
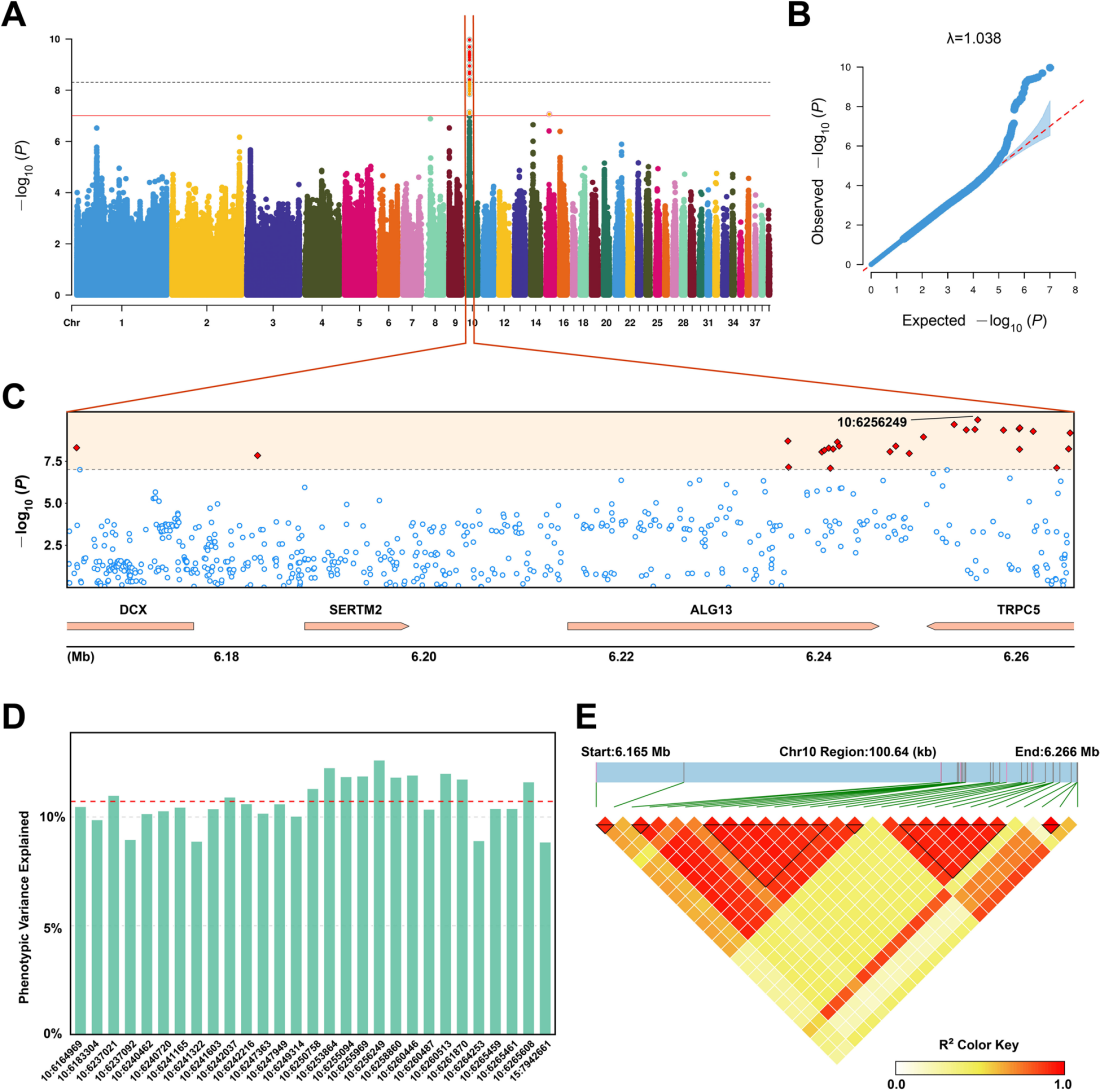
GWAS of Pekin duck genetics and FCR variation. **A** Manhattan plot of host genomic associations with FCR variation. The horizontal black and red lines indicate genome-wide significance (*P* = 4.92 × 10^−9^; 0.05/N_SNP_) and suggestive significance (*P* = 9.84 × 10^−8^; 1/10,159,633) thresholds. Each point represents an SNP. **B** Q-Q plot of host genomic associations with FCR variation. **C** Close-up plot of 6.164–6.266 Mb in chromosome 10. **D** Distribution of PVE value for FCR by the significant FCR-associated SNPs. The horizontal red line represents the mean value of PVE. **E** LD plot of significant FCR-associated SNPs. The black triangles indicate LD blocks

To elucidate the genetic architecture underlying FCR, we first estimated the contribution of host genetics, and obtained that the heritability ($h^{2}$) for the FCR was 0.23 (*P*_LRT_ = 2.53 × 10^–2^), indicating that host genetics have a substantial role in the determination of feed efficiency. Afterwards, we performed GWAS using whole-genome resequencing data and the FCR phenotype, and identified a strong signal of 29 FCR-associated SNPs (*P* < 9.84 × 10^−8^) (Fig. 2A, B and Table S1). All these SNPs clustered on chromosome 10 except for one on chromosome 15. The phenotypic variation explained (PVE) value for FCR by these significant SNPs ranged from 8.8% to 12.6%, with an average contribution of 10.7% (Fig. 2D), suggesting that multiple loci with moderate effects collectively contribute to feed efficiency variation in Pekin ducks. Focus on these FCR-associated SNPs, the regional association plot further pinpointed the 6.164–6.266 Mb region of chromosome 10, where 16 SNPs, 11 SNPs, and 1 SNP were located on genes *TRPC5*, *ALG13*, and *DCX*, respectively (Fig. 2C). The remaining 1 SNP locus was located on the intergenic region between the *DCX* and *SERTM2* genes. To fine‐map the associated locus, we computed pairwise linkage disequilibrium (LD) among SNPs within the association peak region using the *r*^2^ metric (Fig. 2E). LD block analysis based on *r*^2^ identified 6 discrete haplotype blocks within the locus, with block lengths of approximately 18.3 kb, 71 bp, 8.1 kb, 3.1 kb, 5.9 kb, and 2 bp. Notably, one major block (~18.3 kb, located on gene *ALG13*) and a secondary block (~8.1 kb, located on gene *TRPC5*) harbor the majority of tightly linked SNPs, suggesting preserved haplotypes and limited historical recombination within these regions. In contrast, the remaining micro-blocks (2–71 bp) represent fragmented LD segments likely shaped by frequent recombination or allele turnover. This LD architecture indicates a core functional haplotype flanked by smaller recombination-derived segments, supporting the presence of a stable genetic module in this genomic region in modulating FCR in Pekin ducks.

### Association between gut microbial communities and host genetics

**Fig. 3:**
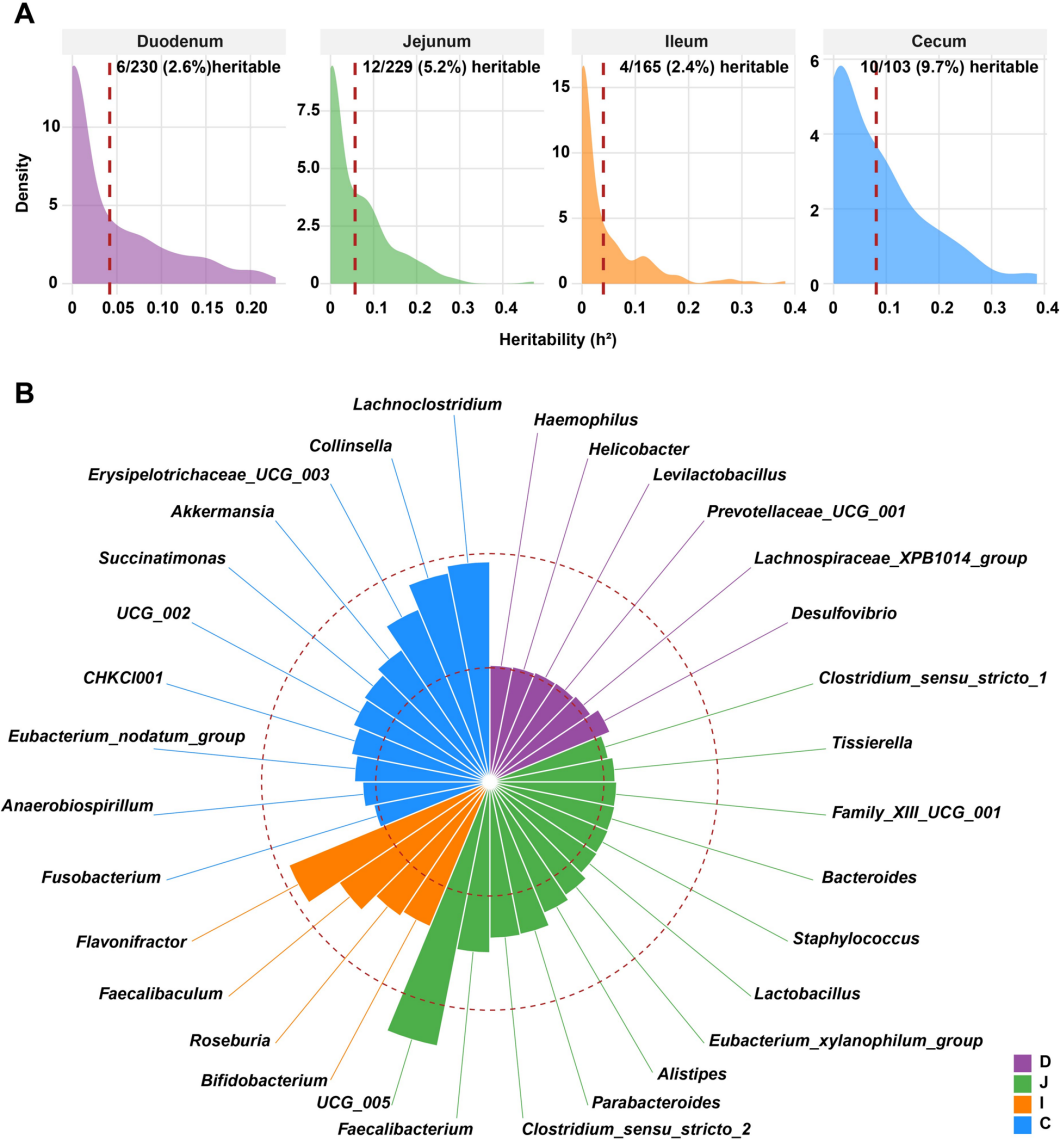
Microbial heritability distribution in different intestinal segments of Pekin ducks. **A** Percentage of heritable microbial genera in different intestinal segments of Pekin ducks. The red dashed line indicates mean heritability. The microbial taxa meet the criteria of $h^{2}$ > 0.2 and *P*_LRT_ < 0.05 was identified as significantly heritable taxa. **B** Heritability estimates for significantly heritable taxa in different intestinal segments of Pekin ducks. The inner red dashed circle represents $h^{2}$ of 0.2, and the outer one represents $h^{2}$ of 0.4

To investigate the host genetic contribution to gut microbial variation across intestinal regions, $h^{2}$ of microbiota was estimated for each genus in four intestinal segments. Overall, the estimated heritability values were low to moderate, with 6 (2.6%), 12 (5.2%), 4 (2.4%), and 10 (9.7%) microbial taxa identified as significantly heritable ($h^{2}$ > 0.2 and *P*_LRT_ < 0.05) in the duodenum, jejunum, ileum, and cecum, respectively (Fig. 3A and Supplementary Table S2). Notably, the cecum exhibited the highest proportion of heritable taxa, suggesting stronger host genetic effect over microbial composition in this fermentation-rich segment of the gut. Among these heritable taxa, *Desulfovibrio* ($h^{2}$ = 0.23, *P*_LRT_ = 3.50 × 10^–2^), *UCG_005* ($h^{2}$ = 0.47, *P*_LRT_ = 7.61 × 10^–6^), *Flavonifractor* ($h^{2}$ = 0.38, *P*_LRT_ = 8.89 × 10^–4^), and *Lachnoclostridium* ($h^{2}$ = 0.39, *P*_LRT_ = 7.45 × 10^–4^) were the microorganisms with the highest heritability in duodenum, jejunum, ileum, and cecum, respectively (Fig. 3B and Supplementary Table S2). To further identify specific genetic loci associated with these heritable taxa, the mGWAS was performed using genome data and the relative abundance (CLR transformed) of taxa. Significant SNP–microbe associations were detected in four intestinal segments. Specifically, 3, 1, 3, 11 SNPs were associated with genera in the duodenum, jejunum, ileum, cecum, respectively (Table 1). These findings indicate that host genetic influences on the gut microbiota are region-specific, with a greater number of associated SNPs observed in the cecum compared with the small intestine.

**Table 1:** Detail of SNPs associated with microbiota in four intestinal segments

Microbiota	SNP (Chromosome: Locus)
*Haemophilus* (D)	31:1159831
*Helicobacter* (D)	2:159554265
*Lachnospiraceae_XPB1014_group* (D)	1:3199141
*Bacteroides* (J)	28:2376757
*Flavonifractor* (I)	3:121046904
*Roseburia* (I)	2:38470374; 5:58100785
*Anaerobiospirillum* (C)	3:3642003
*CHKCI001* (C)	1:37000378; 3:15345521; 3:15345523; 3:15345534; 3:15352488; 3:15352493
*UCG_002*(C)	20:2057175; 20:2060988; 20:2080425; 20:2080431

D, J, I, C denote duodenum, jejunum, ileum, and cecum, respectively

**Fig. 4:**
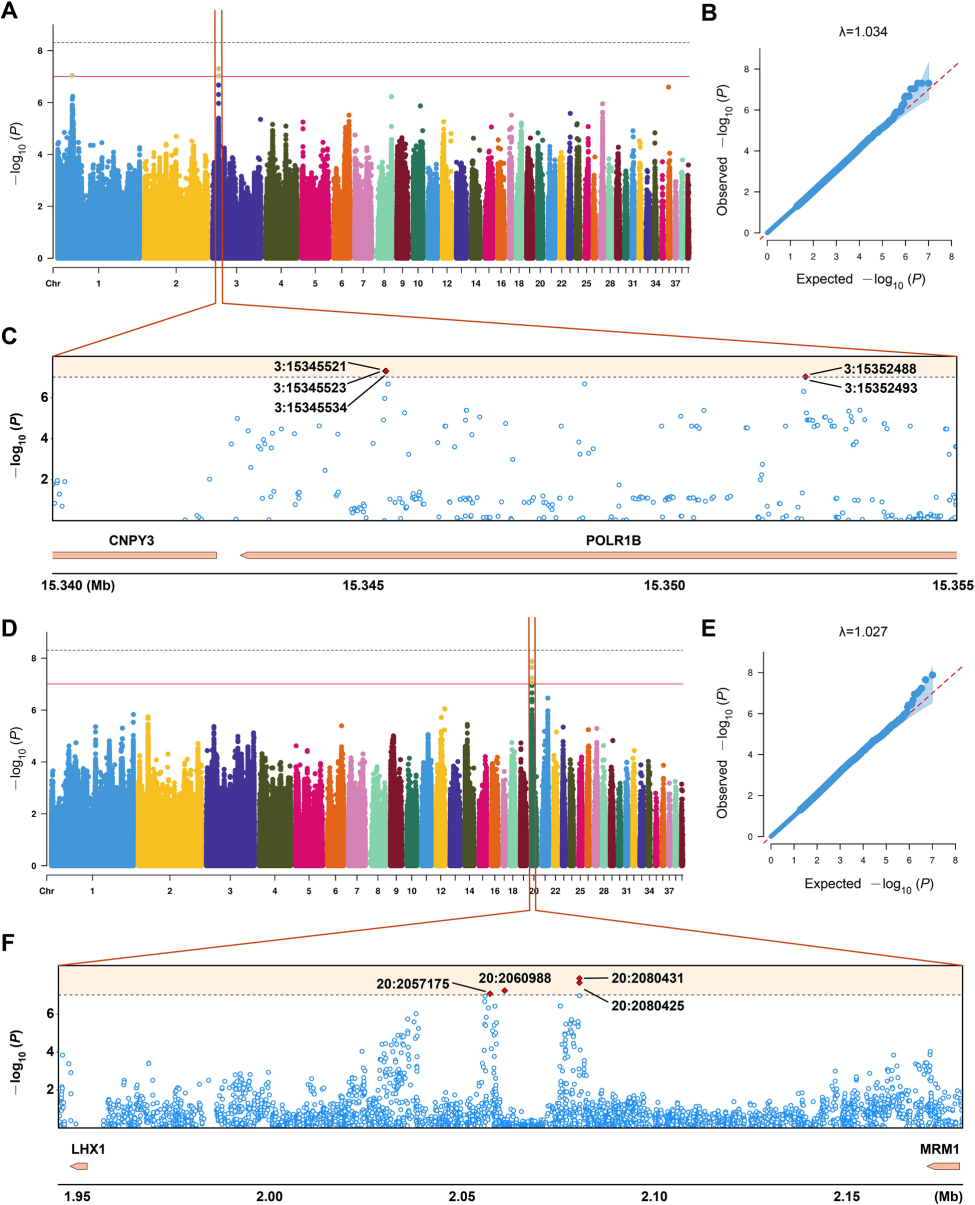
Microbial genome-wide association study of Pekin duck genetics and microbial features variation in cecum. **A** Manhattan plot of host genomic associations with *CHKCI001* feature. The horizontal black and red lines indicate genome-wide significance (*P* = 4.92 × 10^−9^; 0.05/N_SNP_) and suggestive significance (*P* = 9.84 × 10^−8^; 1/10,159,633) thresholds. Each point represents an SNP. **B** Q-Q plot of host genomic associations with *CHKCI001* feature. **C** Close-up plot of 15.340–15.355 Mb in chromosome 3. **D** Manhattan plot of host genomic associations with *UCG_002* feature. The horizontal black and grey lines indicate genome-wide significance (*P* = 4.92 × 10^−9^; 0.05/N_SNP_) and suggestive significance (*P* = 9.84 × 10^−^^8^; 1/10,159,633) thresholds. Each point represents an SNP. **E** Q-Q plot of host genomic associations with *UCG_002* feature. **F** Close-up plot of 1.94–2.18 Mb in chromosome 20

To further characterize the genetic architecture underlying cecal microbiota, we focused on the 2 taxa (*CHKCI001* and *UCG_002*) that exhibited significant SNP associations in this intestinal segment. According to the suggestive significant threshold of *P* < 9.84 × 10^−8^, we identified 6 and 4 SNP loci associated with *CHKCI001* and *UCG_002*, respectively (Fig. 4A–F). A regional association plot highlighted 5 significant *CHKCI001-*associated SNPs were located on the intron of gene *POLR1B* on chromosome 3, of which 3 SNPs (3:15345521, 3:15345523, 3:15345534) all have the lowest *P*-value at 4.96 × 10^−8^ (Fig. 4C). For *UCG_002*, 4 significantly associated SNPs were all located on the intergenic region, with the 2 nearby genes being *LHX1* and *MRM1* (Fig. 4F). The top-associated SNP was 20:2080431 with the *P*-value of 1.32 × 10^−8^. Together, these results demonstrate that specific host genomic regions may influence the abundance of heritable microbial taxa such as *CHKCI001* and *UCG_002*.

### Identification of FCR-associated microbiota affected by host genetics

**Fig. 5:**
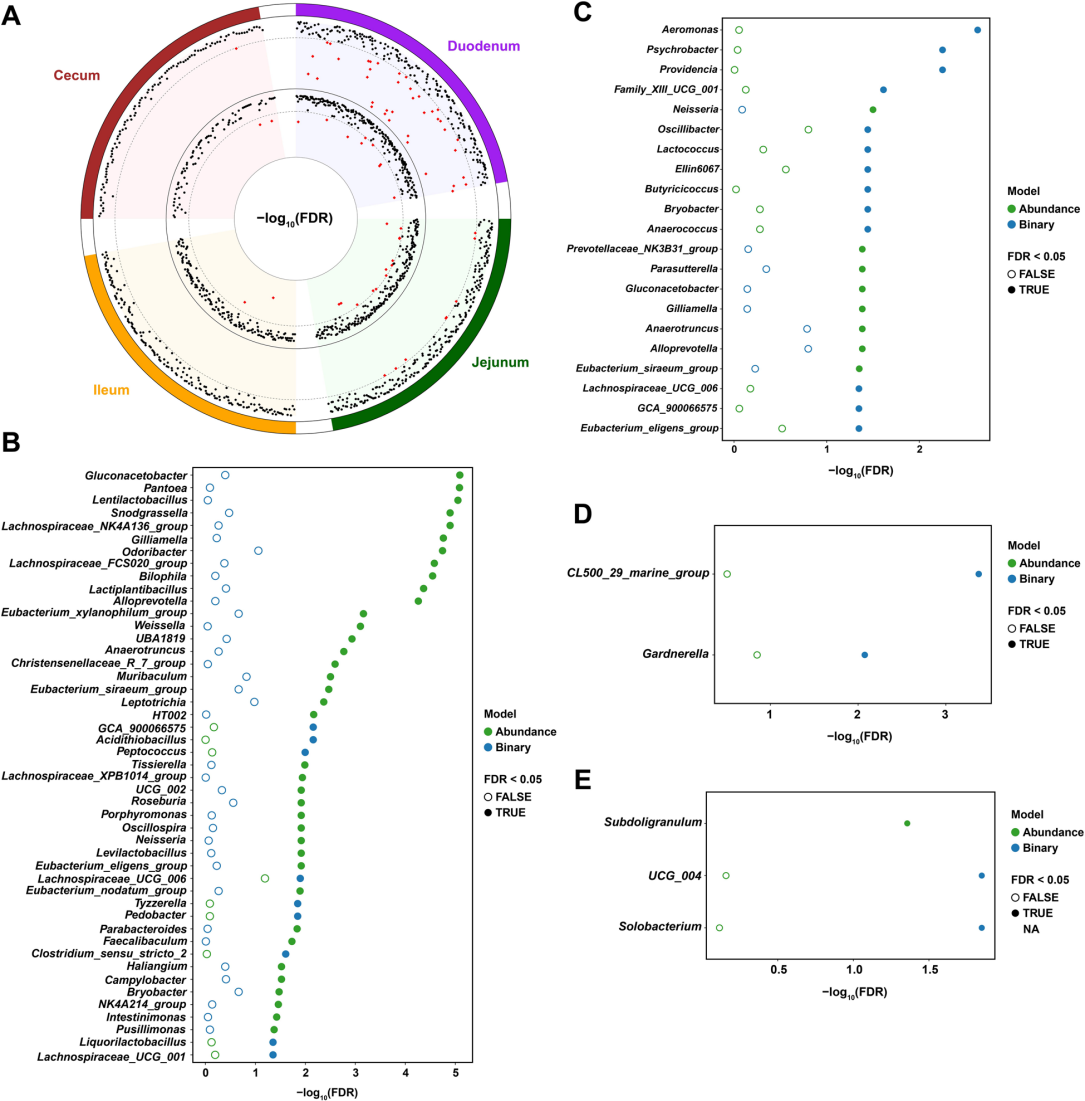
Feed efficiency-related microorganisms. **A** The circular association plot of two-part model among four intestinal segments. The inner circle represents the binary part model, while the outer one denotes the abundance part model. Each point represents one microbe, of which red ones mean the taxa showing significant differences across genotypes. The dash black lines stand for the significant threshold of FDR = 0.05. **B** Significant taxa obtained from the two-part model in duodenum. **C** Significant taxa obtained from the two-part model in jejunum. **D** Significant taxa obtained from the two-part model in ileum. **E** Significant taxa obtained from the two-part model in cecum

Specific microbial taxa can influence host FCR. A two-part model was first applied to identify the microbial taxa significantly associated with FCR across four gut segments. As visualized in the circular association plot (Fig. 5A), a substantial number of taxa exhibiting significant associations with FCR were identified through either the abundance model or binary model. Notably, the outer ring (abundance model) detected a greater number of significant associations than the inner ring (binary model) in the duodenum and jejunum, while the opposite trend in the ileum and cecum. Subsequent segmentation of these data by gut region quantified this finding: the jejunum contained the highest number of associated genera (number = 21), followed by the duodenum (number = 16), with far fewer hits in the cecum (number = 3) and ileum (number = 2) (Fig. 5B–E). These results suggested that the associations between microbial taxa and FCR exhibited distinct spatial patterns.

**Fig. 6:**
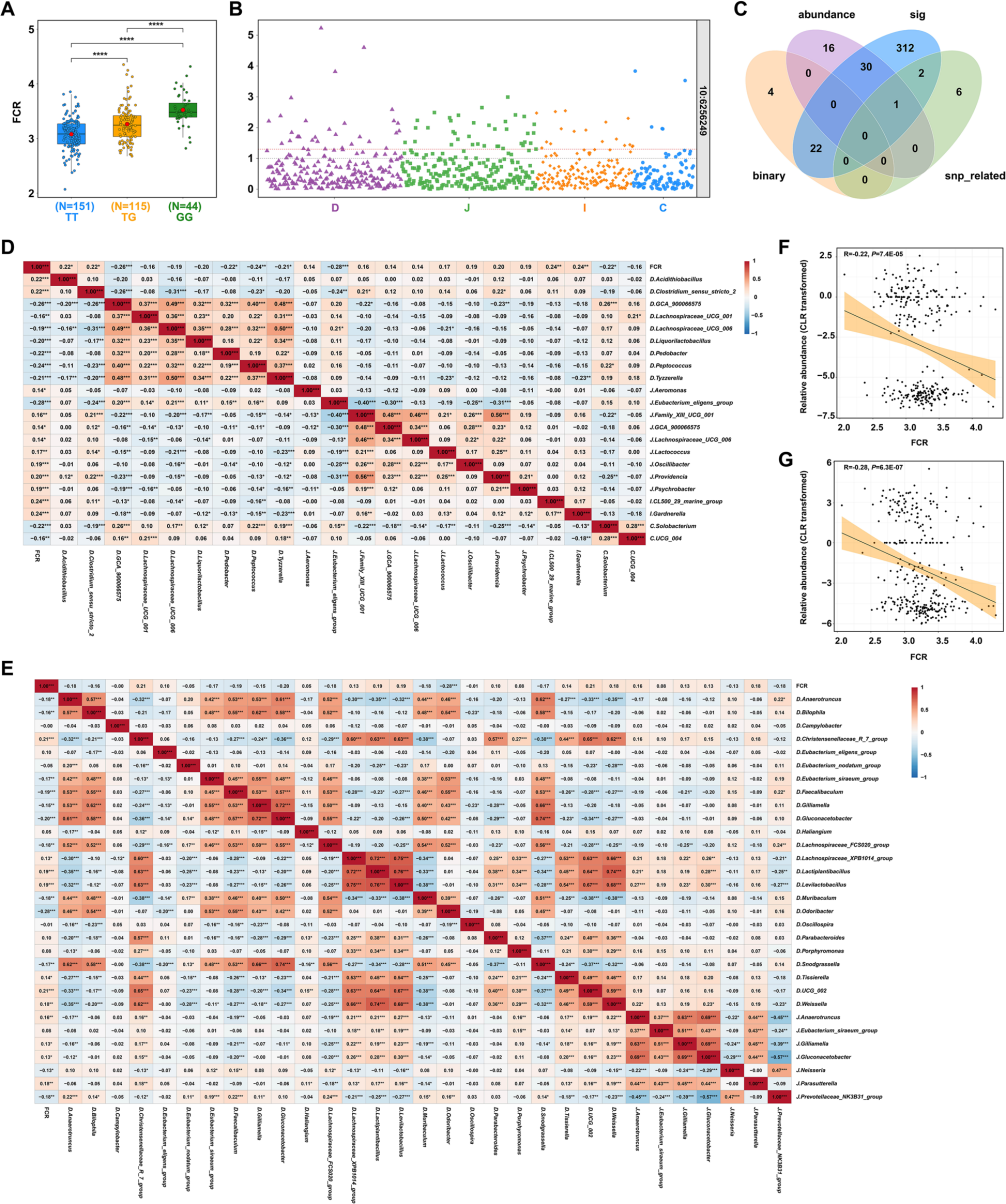
FCR-associated microbiota affected by host genetics. **A** Comparison of FCR among genotypes within the FCR-related SNP Chr10:6256249. Each point represents a duck. The center red point indicates the mean value in the corresponding genotype. ^****^P < 0.0001. **B** Comparison of microbial abundance (CLR transformed) between genotypes within the FCR-related locus Chr10:6256249. Each point represents a duck. The dashed red and gray lines indicate *P* value = 0.05 and 0.1, respectively. **C** Intersection of the key taxa from all methods. “Binary” means taxa identified by the binary model; “abundance” represents taxa identified by the abundance model; “sig” stands for genotype-associated taxa; “snp” denotes taxa with mGWAS-identified SNPs. **D** Correlation of FCR and the taxa which overlapped between genotype-associated taxa and taxa identified by the binary model. **E** Correlation of FCR and the taxa which overlapped between genotype-associated taxa and taxa identified by the abundance model. **F** Correlation between the *Solobacterium* in the cecum and FCR. **G** Correlation between the *Eubacterium_eligens_group* in the jejunum and FCR

Afterwards, to identify the FCR-associated microbiota affected by host genetics, we further selected significant FCR-associated SNPs and compared FCR values among different genotype groups. At the most significant locus (Chr10:6256249; *P*_LRT_ = 1.07 × 10^−^^10^, PVE = 12.6%), ducks carrying the TT genotype exhibited substantially lower FCR values compared with TG (|ΔFCR| = 0.18, *P* < 0.0001) and GG genotypes (|ΔFCR| = 0.44, *P* < 0.0001) (Fig. 6A), indicating that the T-to-G substitution markedly increased FCR and thereby impaired FE. Consistent trends were observed across most other loci, where allelic substitutions were associated with increased FCR and reduced FE, except for the locus Chr15:7942661 (Fig. S1–S4). At this site, a C/T transversion showed a specific pattern: ducks with CC or CT genotypes (no significant difference between the two) were more feed efficient than those with the TT genotype (Fig. S4). Moreover, the differences in the abundance of each microbiota were analyzed among the different genotypes to further investigate the joint effects of host genotypes and gut microbiota on FCR. At Chr10:6256249 locus, a total of 102 microbial genera reached the significance level, including 32, 46, 19, and 5 genera in duodenum, jejunum, ileum, and cecum, respectively (Fig. 6B). Combining results among different genotypes across all significant loci, a total of 368 significant microorganisms were detected in four intestinal segments.

Integrating the taxa from two-part model results, taxa with mGWAS-identified SNPs, and taxa showing significant differences across genotypes at FCR-associated SNPs, we identified several overlapping taxa emerged across these approaches (Fig. 6C), which were the host genetics-influenced taxa associated with FCR. Specifically, 22 genera overlapped between genotype-associated taxa and taxa identified by the binary model, including 12 positively correlated genera (2 in duodenum; 8 in jejunum; 2 in ileum) and 10 negatively correlated genera (7 in duodenum; 1 in jejunum; 2 in cecum) with FCR (Fig. 6D). Similarly, 31 genera overlapped between genotype-associated taxa and taxa identified by the abundance model, among which 11 genera (7 in duodenum; 4 in jejunum) showed positive correlations and 12 genera (10 in duodenum; 2 in jejunum:) exhibited negative correlations with FCR (Fig. 6E). Of these FCR-associated taxa affected by host genetics, 2 known SCFA-producing microorganisms—*Solobacterium* in the cecum and *Eubacterium_eligens_group* in the jejunum—showed significant negative correlations with FCR (*r* = −0.22 and *r* = −0.28, respectively) (Fig. 6F and G). Together, these findings collectively demonstrate that host genetics contribute to FCR not only through direct effects but also by shaping the gut microbiota in an intestinal-segment-specific manner.

### Explainability of host genetics and gut microbiota for FCR

**Fig. 7:**
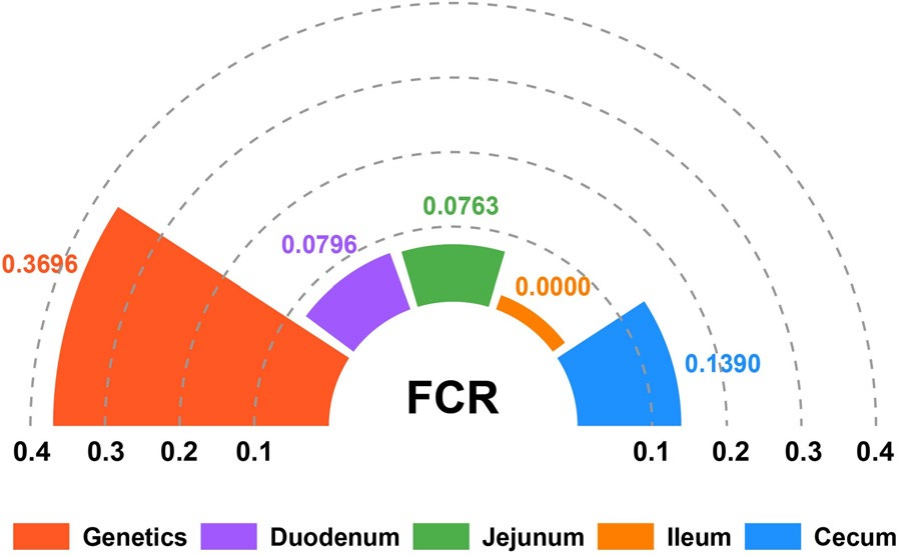
Explainability of host genetics and gut microbiota for FCR

Above results revealed that the host genetics and gut microbiota jointly regulate FCR. To further quantify the contributions of two factors to FCR, MLM analysis was performed to partition the variation in FCR (see Methods). As shown in Fig. 7, host genetics explained the largest proportion of FCR variation (36.96%, *P*_LRT_ = 3.14 × 10^–6^), followed by microbial composition in the cecum (13.90%, *P*_LRT_ = 0.0090), duodenum (7.96%,* P*_LRT_ = 0.0955), and jejunum (7.63%,* P*_LRT_ = 0.2068), whereas the ileum microbiota contributed negligibly (0.00%,* P*_LRT_ = 0.5). These results highlight the predominant role of host genetic background in determining FCR while also demonstrating region-specific microbial influences, particularly from the cecum and proximal small intestine.

## Discussion

In recent years, enhancing FE has emerged as a major breeding goal in Pekin ducks. Previous studies have established FE as a complex trait shaped by both host genetics and gastrointestinal microbiota [9, 31, 47]. However, it remains unclear whether and how host genetics interact with the gut microbiota to modulate FE in Pekin ducks. Elucidating such mechanisms is essential for developing effective strategies to improve FE through breeding and management.

Herein, we first systematically investigated the composition of microbial communities in four GIT regions of ZF and CF ducks via 16S rRNA sequencing, and found significant spatial heterogeneity along the intestinal segments between two groups. The observed predominance of *Ligilactobacillus* in the duodenum and jejunum aligns with its known function in fermenting carbohydrates and stimulating the production of SCFAs, metabolites that are crucial for maintaining gut health [48–50]. The dominance of *Bacteroides* in the cecum can be explained by its well-characterized role as a mucin-degrading taxon, which relies on a significantly larger set of genes encoding carbohydrate-active enzymes (CAZymes) for breaking down both mucins and dietary polysaccharides in cecum [51–54].

Then we used FCR as a representative indicator of FE, and evaluated its heritability. Aligning with previous findings, our results revealed a moderate heritability for FCR ($h^{2}$ = 0.23) [20, 55, 56]. It is important to note that heritability estimation using REML typically benefits from larger sample sizes for greater precision. Although our estimation (moderate heritability under the moderate sample size) is consistent with previous reports [18, 19, 57–59], the moderate sample size (*n* = 313) may introduce uncertainty. Future studies with larger populations are warranted to validate these findings. Moreover, GWAS revealed key genetic components contributing to FCR variation in Pekin ducks, of which a major block (~18.3 kb, located on *ALG13*) and a secondary block (~8.1 kb, located on *TRPC5*) covered the majority of FCR-associated SNPs, indicating their potential roles in regulating FCR. Interestingly, *ALG13* has been reported to regulate the expression of gamma-aminobutyric acid A receptor (GABA_A_R) [60], which is known as mediator of satiety and body weight regulation in the lateral hypothalamus [61, 62]. *TRPC5* was identified as a promising candidate gene based on its established role in central energy homeostasis. Li et al. [63] reported that conditional deletion of *TRPC5* in oxytocin neurons within the hypothalamus led to hyperphagia, excessive body weight gain, and fat accumulation in mice, highlighting its critical role in appetite suppression and metabolic balance. Similarly, Gao et al. revealed that *TRPC5*-mediated calcium signaling in POMC neurons is essential for leptin- and serotonin-induced anorexigenic responses, suggesting that *TRPC5* contributes to maintaining negative energy balance through neuroendocrine pathways [64]. Consistently, Rode et al. [65] showed that alterations in *TRPC5* channel activity significantly affected body weight gain, reinforcing its involvement in energy metabolism. Although direct evidence for hypothalamic expression in ducks is currently unavailable, both *ALG13* and *TRPC5* are evolutionarily conserved at the protein level. The identified SNPs reside within haplotype blocks containing regulatory elements, supporting their potential role in modulating energy balance pathways. For example, mutation of 10:6255969 and 10:6256249 located on the 3'UTR region of *TRPC5* are predicted to influence the binding of miRNAs. Collectively, these findings suggest that genetic variation in *ALG13* and *TRPC5* can influence FE of ducks by modulating appetite and energy expenditure.

Beyond host genetics, the gut microbiota also serves as a key regulator of FE [9, 66–68]. As the host second genome [69], it actively participates in feed digestion and nutrient absorption [70, 71]. Gut microbial community analysis first revealed distinct spatial microbial patterns, consistent with known duck gut biogeography [33, 72, 73]. Afterwards, microbial source tracking confirmed that a large proportion of cecal taxa originate from yet uncharacterized sources, indicating a highly distinct microbial ecosystem in the Pekin duck cecum, which is consistent with the finding in chicken [9]. We next identified several heritable genera in Pekin ducks, with the cecum showed the highest proportion of heritable taxa and the most number of significant SNP–microbe associations. This finding indicated strongest host genetic influence on microbial composition in this fermentation-active site, aligning with previous chicken studies: Wen et al. [9] identified multiple host genomic loci linked to cecal genera such as *Parabacteroides* and *Megasphaera* in chickens, and Feng et al. [74] reported clear genotype-driven differences in cecal microbiota across chicken lines, collectively underscoring the substantial genetic regulation of cecal microbial communities.

Consistent with our earlier work characterizing spatial heterogeneity in the duck gut microbiota, the current study further identifies FCR-associated taxa that exhibit segment-specific enrichment, underscoring the functional specialization of each intestinal region. For example, the significantly negative association between *Solobacterium* and FCR is observed in the cecum, whereas no such association is found in other segments, suggesting *Solobacterium* is niche-specific. This cecal specialization aligns with findings in porcine models, where *Solobacterium* is mainly enriched in the hindgut, responding to soluble fiber intake and fermentation [75, 76].

Furthermore, to investigate the joint effects of host genotypes and gut microbiota on FE, we integrated the taxa with mGWAS-identified SNPs, the taxa from two-part model results, and taxa showing significant differences across genotypes at FCR-associated SNPs, and found several overlapping taxa across these approaches. Among the host genetics-influenced taxa associated with FCR, 2 microorganisms negatively correlated with FCR—*Solobacterium* in the cecum and *Eubacterium_eligens_group* in the jejunum—have been demonstrated to be involved in FE regulation. Specifically, *Solobacterium* is involved in the synthesis of SCFA and biohydrogenation of fatty acids [77, 78], and affect specific metabolic pathways [79] to enable host to obtain more energy, thereby improving FE. For the other key taxa, Bergamaschi et al. [80] found a positive association between the abundance of *Eubacterium_eligens_group* and FE [80]. In swine, low-protein diets reduced the abundance of *Eubacterium_eligens_group* in the hindgut, impaired microbial fermentation capacity, and decreased SCFA production, ultimately leading to compromise nutrient digestibility and diminish FE [81].

Taken together, our findings demonstrate that FE in Pekin ducks is co-regulated by host genetics and the gut microbiota. MLM analysis further quantified their respective contributions, not only revealing host genetics as the dominant factor—explaining 36.96% of FCR variation, but also highlighting the particular importance of the cecum microbiota among four intestinal segments.

## Conclusion

In summary, our study demonstrates that feed efficiency in Pekin ducks is jointly influenced by host genetics and gut microbiota, with host genetic factors exerting the predominant effect. High-depth whole-genome sequencing and GWAS identified key candidate genes, including *ALG13* and *TRPC5*, which may regulate feed efficiency through appetite control and energy metabolism. Meanwhile, gut microbial communities exhibited distinct spatial patterns along the intestinal tract, with specific taxa such as *Solobacterium* in the cecum and *Eubacterium_eligens_group* in the jejunum showing significant associations with FCR, likely via enhanced short-chain fatty acid production and improved energy harvest. These findings highlight the intricate interplay between host genetics and gut microbiota in shaping feed efficiency and provide valuable targets for genetic selection and microbiota-based interventions aimed at improving feed utilization in Pekin ducks.

## Supplementary Information

Additional file 1: Fig. S1 Comparison of FCR among genotypes within the FCR related SNPs. A–I represent these SNPs: 10:6164969; 10:6183304; 10:6237021; 10:6237092; 10:6240462; 10:6240720; 10:6241165; 10:6241322; and 10:6241603, respectively. Fig. S2 Comparison of FCR among genotypes within the FCR related SNPs. A–I represent these SNPs: 10:6242037; 10:6242216; 10:6247363; 10:6247949; 10:6249314; 10:6250758; 10:6253864; 10:6255094; and 10:6255969, respectively. Fig. S3 Comparison of FCR among genotypes within the FCR related SNPs. A–I represent these SNPs: 10:6258860; 10:6260446; 10:6260487; 10:6260513; 10:6261870; 10:6264253; 10:6265459; 10:6265461 and 10:6265608, respectively. Fig. S4 Comparison of FCR among genotypes within the FCR related SNP 15:7942661.

Additional file 2: Table S1. FCR-associated SNPs on chromosome 10.

Additional file 3: Table S2. Heritability estimates for significantly heritable taxa in different intestinal segments of Pekin ducks.

## Data Availability

The WGS and 16S rRNA datasets generated and analyzed during the current study are available in Genome Sequence Archive in National Genomics Data Center (GSA: CRA033474 and CRA026543), respectively.

## Abbreviations

ANOVA: Analysis of variance
ASVs: Amplicon sequence variants
BH: Benjamini-Hochberg
BWG: Body weight gain
CLR: Centered log-ratio
DDL: Duck-derived lactic acid bacteria
FCR: Feed conversion ratio
FE: Feed efficiency
FI: Feed intake
GABA_A_R: Gamma-aminobutyric acid A receptor
GRM: Genetic relationship matrix
GWAS: Genome-wide association studies
LD: Linkage disequilibrium
LRT: Likelihood ratio test
mGWAS: Microbiome genome-wide association studies
MLM: Mixed linear model
MRM: Microbial relationship matrix
MWAS: Microbiome-wide association studies
NMDS: Non-metric multidimensional scaling
PCA: Principal component analysis
PCs: Principal components
PVE: Phenotypic variation explained
QTLs: Quantitative trait loci
REML: Restricted maximum likelihood
SCFAs: Short-chain fatty acids
SNPs: Single nucleotide polymorphisms
