# Supplementary material for: Integrated analyses of host genetics and gut microbiota provide mechanistic insights into feed efficiency in ducks
Source: J Anim Sci Biotechnol. 2026 May 19;17:97. doi: 10.1186/s40104-026-01410-1 (PMC13185347; doi:10.1186/s40104-026-01410-1)
Supplement: Supplementary file 1 — Additional file 1: Fig. S1 Comparison of FCR among genotypes within the FCR related SNPs. A–I represent these SNPs: 10:6164969; 10:6183304; 10:6237021; 10:6237092; 10:6240462; 10:6240720; 10:6241165; 10:6241322; and 10:6241603, respectively. Fig. S2 Comparison of FCR among genotypes within the FCR related SNPs. A–I represent these SNPs: 10:6242037; 10:6242216; 10:6247363; 10:6247949; 10:6249314; 10:6250758; 10:6253864; 10:6255094; and 10:6255969, respectively. Fig. S3 Comparison of FCR among genotypes within the FCR related SNPs. A–I represent these SNPs: 10:6258860; 10:6260446; 10:6260487; 10:6260513; 10:6261870; 10:6264253; 10:6265459; 10:6265461 and 10:6265608, respectively. Fig. S4 Comparison of FCR among genotypes within the FCR related SNP 15:7942661. [file 40104_2026_1410_MOESM1_ESM.pdf]

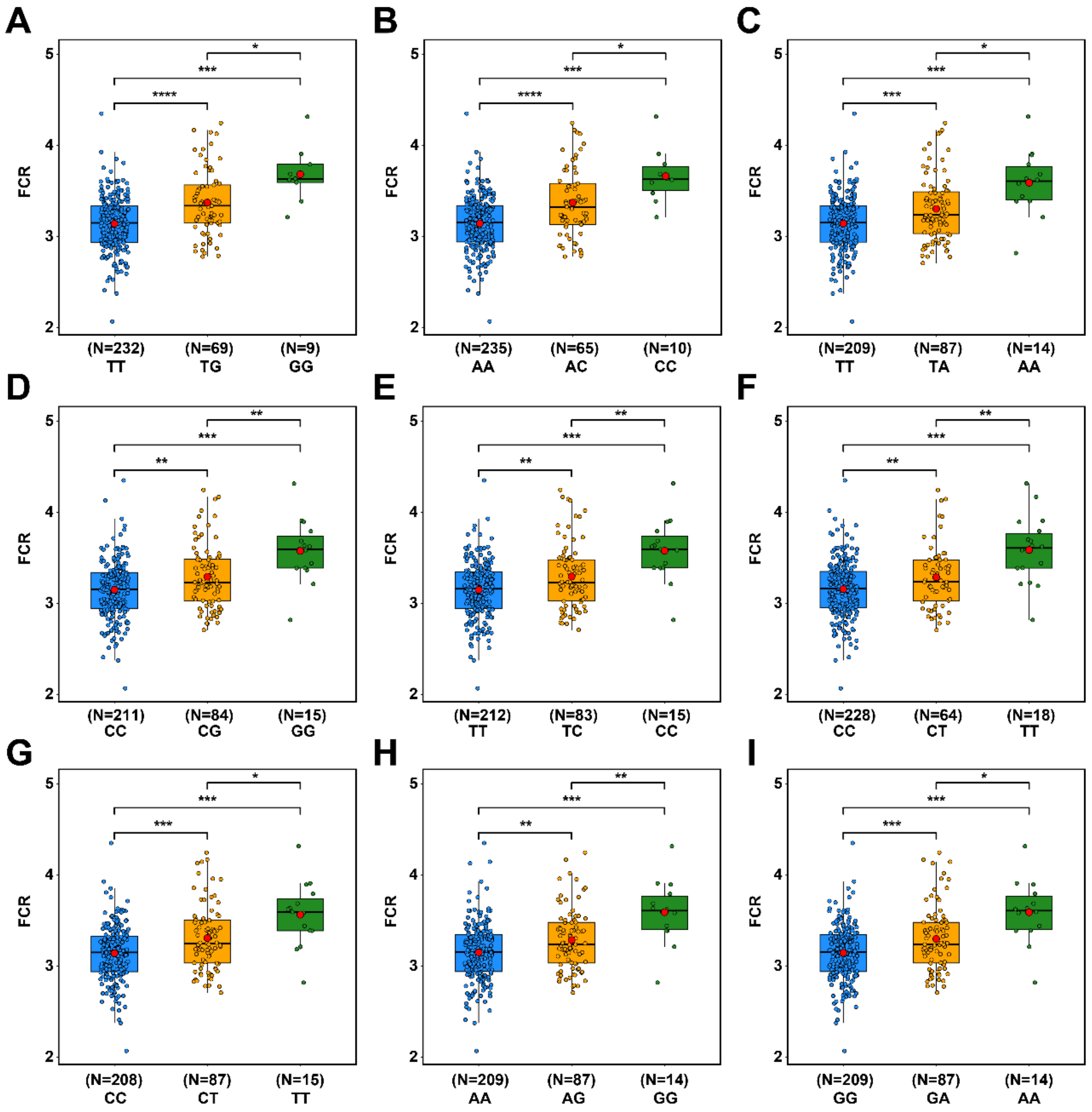

Figure S1 Comparison of FCR among genotypes within the FCR-related SNPs. (A)-(I) represent these SNPs: 10:6164969; 10:6183304; 10:6237021; 10:6237092; 10:6240462; 10:6240720; 10:6241165; 10:6241322; and 10:6241603, respectively.

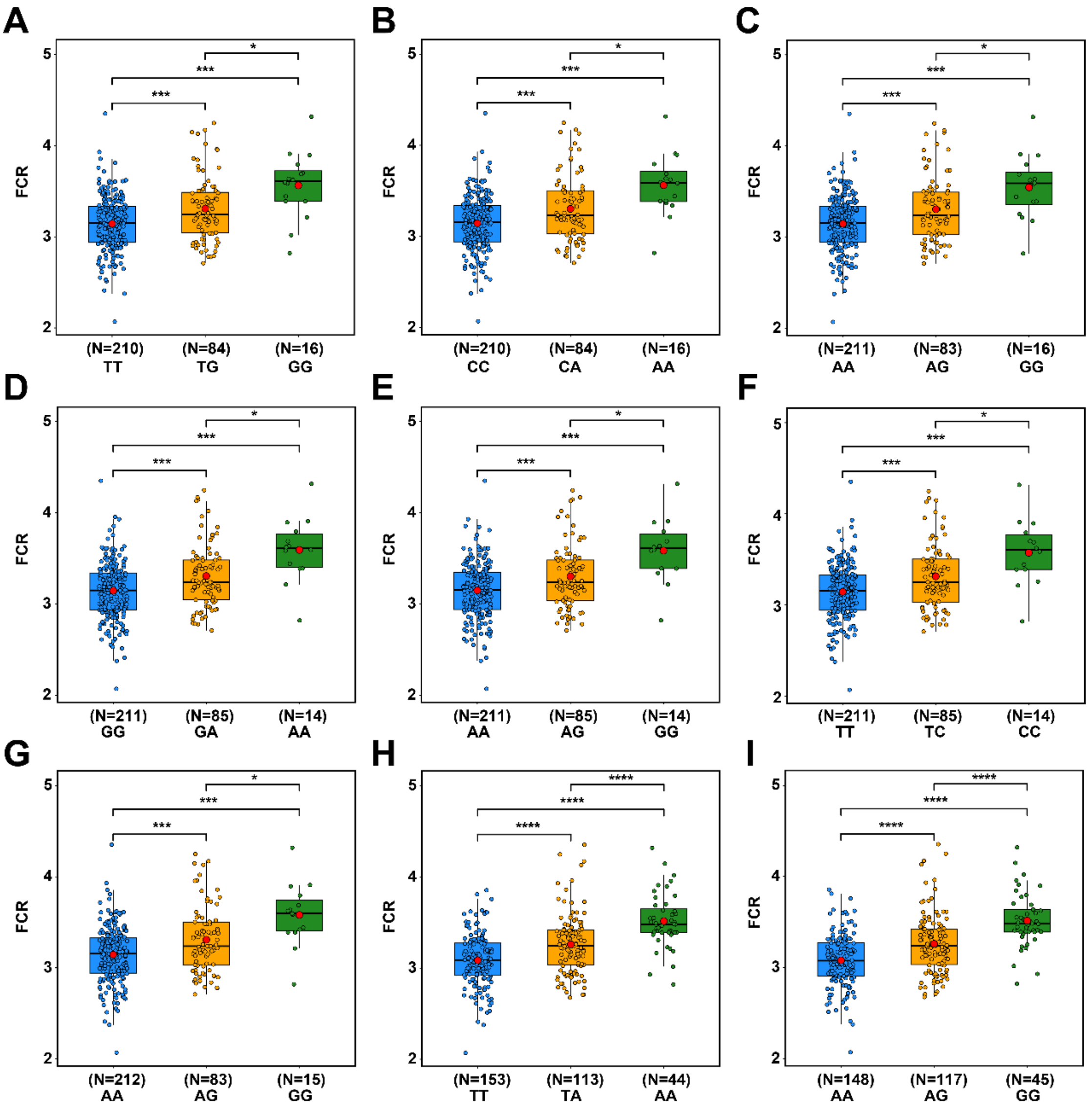

Figure S2 Comparison of FCR among genotypes within the FCR-related SNPs. (A)-(I) represent these SNPs: 10:6242037; 10:6242216; 10:6247363; 10:6247949; 10:6249314; 10:6250758; 10:6253864; 10:6255094; and 10:6255969, respectively.

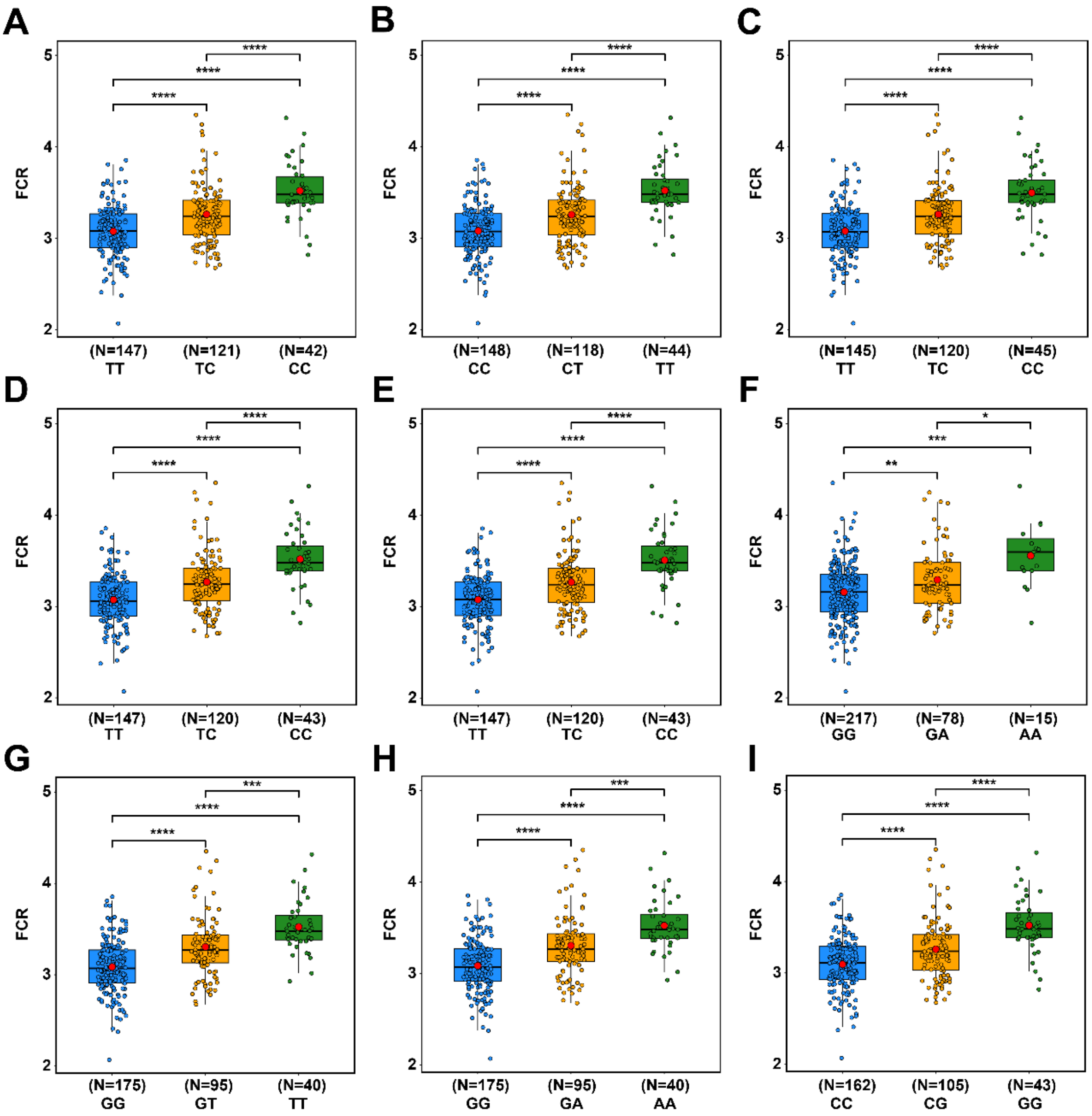

Figure S3 Comparison of FCR among genotypes within the FCR-related SNPs. (A)-(I) represent these SNPs: 10:6258860; 10:6260446; 10:6260487; 10:6260513; 10:6261870; 10:6264253; 10:6265459; 10:6265461; and 10:6265608, respectively.

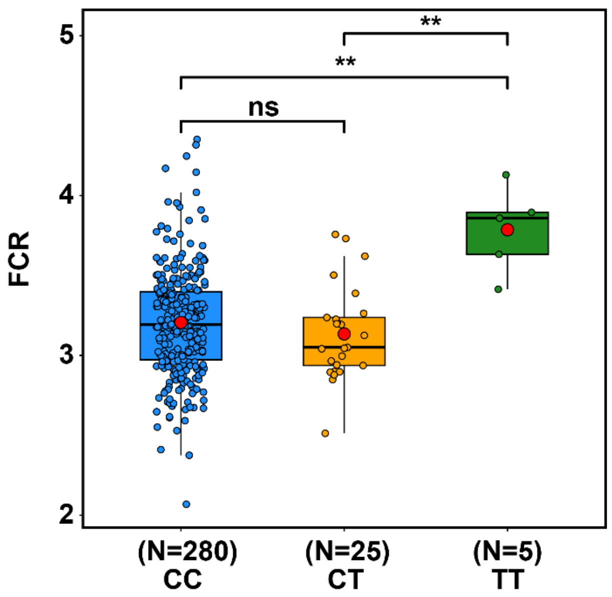

Figure S4 Comparison of FCR among genotypes within the FCR-related SNP 15:7942661.
